# Seroprevalence survey of anti-SARS-CoV-2 antibody and associated factors in South Africa: Findings of the 2020–2021 population-based household survey

**DOI:** 10.1371/journal.pgph.0002358

**Published:** 2023-09-25

**Authors:** Sizulu Moyo, Leickness C. Simbayi, Khangelani Zuma, Nompumelelo Zungu, Edmore Marinda, Sean Jooste, Shandir Ramlagan, Mirriam Fortuin, Beverley Singh, Musawenkosi Mabaso, Tarylee Reddy, Whadi-ah Parker, Inbarani Naidoo, Samuel Manda, Ameena Goga, Nobubelo Ngandu, Cherie Cawood, Penny L. Moore, Adrian Puren

**Affiliations:** 1 Human Sciences Research Council, Pretoria, South Africa; 2 School of Public Health and Family Medicine, University of Cape Town, Cape Town, South Africa; 3 Department of Psychiatry & Mental Health, University of Cape Town, Cape Town, South Africa; 4 School of Public Health, University of the Witwatersrand, Johannesburg, South Africa; 5 Department of Psychology, University of Pretoria, Pretoria, South Africa; 6 National Institute for Communicable Diseases of the National Health Laboratory Service, Johannesburg, South Africa; 7 Biostatistics Research Unit (TR and SMa)/ HIV and other Infectious Diseases Research Unit (AG and NN), South African Medical Research Council, Cape Town, South Africa; 8 Department of Paediatrics and Child Health, University of Pretoria, Pretoria, South Africa; 9 Epicentre, Randburg, South Africa; 10 SAMRC Antibody Immunity Research Unit, Division of Virology and Immunology, University of the Witwatersrand, Johannesburg, South Africa; 11 Division of Virology, School of Pathology, University of the Witwatersrand Medical School, Johannesburg, South Africa; Fundacao Oswaldo Cruz, BRAZIL

## Abstract

Population-based serological testing is important to understand the epidemiology and estimate the true cumulative incidence of severe acute respiratory syndrome coronavirus 2 (SARS-CoV-2) to inform public health interventions. This study reports findings of a national household population SARS-CoV-2 serosurvey in people 12 years and older in South Africa. This cross-sectional multi-stage random stratified cluster survey undertaken from November 2020 to June 2021 collected sociodemographic data, medical history, behavioural data, and blood samples from consenting participants. The samples were tested for SARS-CoV-2 antibodies using the Roche ElecsysAnti-SARS-CoV-2 chemiluminescence immunoassay (CLIA) Total Antibody Test. The survey data were weighted by age, race, sex, and province with final individual weights benchmarked against the 2020 mid-year population estimates and accounted for clustering. Descriptive statistics summarize the characteristics of participants and seroprevalence. Logistic regression analyses were used to identify factors associated with seropositivity. From 13290 survey participants (median age 33 years, interquartile range (IQR) 23–46 years), SARS-CoV-2 seroprevalence was 37.8% [95% Confidence Interval (CI) 35.4–40.4] and varied substantially across the country’s nine provinces, and by sex, age and locality type. In the final adjusted model, the odds of seropositivity were higher in women than in men [aOR = 1.3 (95% CI: 1.0–1.6), p = 0.027], and those living with HIV (self-report) [aOR = 1.6 (95% CI: 1.0–2.4), p = 0.031]. The odds were lower among those 50 years and older compared to adolescents 12–19 years old [aOR = 0.6 (95% CI: 0.5–0.8), p<0.001] and in those who did not attend events or gatherings [aOR = 0.7 (95% CI: 0.6–1.0), p = 0.020]. The findings help us understand the epidemiology of SARS-CoV-2 within different regions in a low-middle-income country. The survey highlights the higher risk of infection in women in South Africa likely driven by their home and workplace roles and also highlighted a need to actively target and include younger people in the COVID-19 response.

## Introduction

The coronavirus disease 2019 (COVID-19) caused by the novel severe acute respiratory syndrome coronavirus 2 (SARS-CoV-2) led to one of the biggest public health challenges globally, including in South Africa [1–3]. In the first week of March 2020, South Africa reported its first case of COVID-19 and has since reported one of the highest infections and deaths in Africa [3, 4]. As the number of cases increased, the government declared a national state of disaster against COVID-19 in March 2020 [5]. The country’s response followed international recommendations and ranged from an initial strict lockdown in March 2020 to more relaxed lockdown levels overtime until towards the end of 2021, together with non-pharmaceutical interventions of social distancing, hand washing or sanitizing, and wearing of face masks or face coverings in public spaces, and then phased vaccination roll out from February 2021 [6, 7]. Lockdowns included work-from-home and school closures. However, cases continued to increase indicating ongoing transmission and a need for an improved response underpinned by a better understanding of the epidemiology of the pandemic in the country.

With most infections asymptomatic or presenting mild symptoms [8–10], surveillance of laboratory-confirmed cases detected by reverse transcription polymerase chain reaction (rRT-PCR) assays, or by Antigen-detecting rapid diagnostic tests (Ag-RDTs)) only captures persons who are tested. This represents only a fraction of all cases, thus underestimating the true extent of infection [11, 12]. Serological tests to detect anti-SARS-CoV-2 antibodies provide a better estimate of the true burden of SARS-CoV-2 infection in the general population enabling more effective implementation of infection control and prevention policies [8, 12, 13]. Between March 2020 and January 2021, data from subnational serological studies and from blood donors in metropolitan areas of South Africa using different serological assays showed infection estimates ranging from 31% to 62%, higher than from PCR and antigen testing based cases [14–17]. However, these studies provided limited insights into representative sex, age, national and provincial estimates for most of the country’s nine provinces, and the different locality types of urban areas, traditional rural areas and farms as defined by Statistics South Africa [18], and this represented a critical gap in data needed to respond more appropriately to the pandemic in the country.

Therefore, we undertook a nationally representative survey to understand the epidemiology of SARS-CoV-2 infections and risk factors for seropositivity to provide information to support public health responses at the time and for similar epidemics in the future. Our study was undertaken before national vaccination roll out and before vaccines for adolescents had received regulatory clearance, and provided an opportunity to determine pre-vaccination estimates of infections in the country.

## Methods

### Study design and sampling

The study was a cross-sectional multi-stage stratified cluster population-based household seroprevalence survey conducted in all nine provinces of South Africa. There were two rounds of data collection from November 2020 to June 2021, and these coincided with periods when the original SARS COV-2 (Wuhan D614G) virus strain and the Beta variant respectively were circulating in the country. The survey targeted all locality types (urban areas, rural formal and rural informal areas) within the selected geographic small area layers (SALs) stratified by province. The target population was all people aged 12 years and older living in households within the selected survey SALs.

The sample size calculation was adapted from the 2005 WHO EPI Vaccination Coverage Cluster Survey reference manual [19]) and was originally based on the assumption of a SARS COV-2 seroprevalence of 2% in South Africa, with a margin of error of 2%, and a joint (household and individual) response rate of 70% and was powered to provide an estimate for each of the country’s nine provinces and the four metropolitan areas (Cape Town, Johannesburg, eThekwini, Nelson Mandela Bay). As the pandemic evolved the calculation was revised to consider the more realistic SARS COV-2 seroprevalence of 20%, and a response rate of 64%, resulting in a sample size of 12 625 individuals providing a blood specimen for antibody testing projected to achieve precision levels of 1.5% or lower.

The survey collected data using household and individual questionnaires. The individual questionnaires collected data on sociodemographic characteristics, self-reported medical history (COVID-19 symptoms, lung and other chronic conditions including HIV status), COVID-19 related knowledge, attitudes, risk factors, and behavioural data. The questionnaires were administered by trained fieldworkers and captured electronically using Research Electronic Data Capture (RedCap) [20]. Survey trained nurses and phlebotomists collected venous blood samples from consenting participants. The samples were stored and transported under cold chain conditions to the laboratory within 24 hours of collection.

### Antibody testing

Testing for SARS-CoV-2 antibodies was undertaken at the Centre for HIV and STIs at the National Institute for Communicable Disease (NICD), in Johannesburg using the Roche Elecsys Anti-SARS-CoV-2 (CLIA, Total Antibody Test, Roche Diagnostics GmbH, Sandhofer Strasse, Mannheim a qualitative immunoassay that detects total antibodies, including IgG, IgM and IgA to SARS-CoV-2 in human serum or plasma. The assay has a reported sensitivity of 96.6% (95% CI 93.4–98.5) after ≥15 days post-symptom onset and maintains seroreactivity and high sensitivity over time post the acute infection stage [21].

### Ethical considerations

The Human Sciences Research Council (HSRC) Research Ethics Committee approved the survey protocol (REC 4/17/06/20). Verbal or written informed consent was required for survey participation. For participants younger than 18 years old, assent by the participant and parental/guardian consent were required.

### Measures

The primary outcome variable was seropositivity on the Roche Elecsys Anti-SARS-CoV-2 antibody assay, dichotomized as SARS-CoV-2 positive and SARS-CoV-2 negative.

Explanatory variables were socio-demographic and COVID-19 related behavioural characteristics, and medical history. Socio-demographic characteristics included age, sex, employment status, and residence locality type (urban areas, rural informal/tribal areas, rural formal/farms). COVID-19 related behaviour characteristics were having left the province/ village/suburb/ township of primary residence in the past 7days, attending an event or gathering in the past 14days, close contact with people outside the home in the past 7days, the number of close contacts outside the home the last time you left home, contact history (contact with someone with suspected/confirmed COVID-19 infection at any time from detection of the first confirmed case of SARS-CoV-2 infection in South Africa), place of contact with this person (health care facility, family setting, workplace, public place, other). Medical history included presence of one or more symptoms of COVID-19 infection in the past 14 days and/or in the past 3 months, having ever tested for SARS-CoV-2 infection, and self-reported diagnoses of one or more any of the following:- past or current tuberculosis, moderate to severe asthma, other chronic lung diseases, hypertension, diabetes, cancer not in full remission, cardiovascular conditions, and other chronic conditions. At the time of the study reverse transcriptase PCR testing was the only testing modality available to confirm COVID-19 infection in South Africa.

### Statistical analysis

The survey data were weighted for age, race, sex, and province with final individual weights benchmarked against 2020 mid-year population estimates of South Africa. Descriptive statistics were used to summarize characteristics of the study participants and SARS-CoV-2 seroprevalence. Categorical variables were compared using Pearson χ2 tests. Bivariate and multivariate logistic regression analyses were used to identify factors associated with SARS-CoV-2 seropositivity. Statistically significant covariates from the bivariate regression analyses were included in the final multivariate logistic regression models. Crude odds ratios (OR) and adjusted odds ratios (aOR) with 95% confidence intervals (CI) are presented, and statistical significance was determined at α = 5%. All regression analyses account for the multistage survey design, at province and SAL level, and the selection of all individuals within a household using weighted logistic regression with cluster robust standard errors. Standard errors are therefore adjusted for the stratification as well as the clustering at each stage of the design. All data analyses were conducted using Stata version 15.0 (StataCorp. 2017.*Stata Statistical Software*: *Release 15*. College Station, TX: StataCorp LLC.) software.

## Results

### Participation and sample characteristics

**Fig 1:**
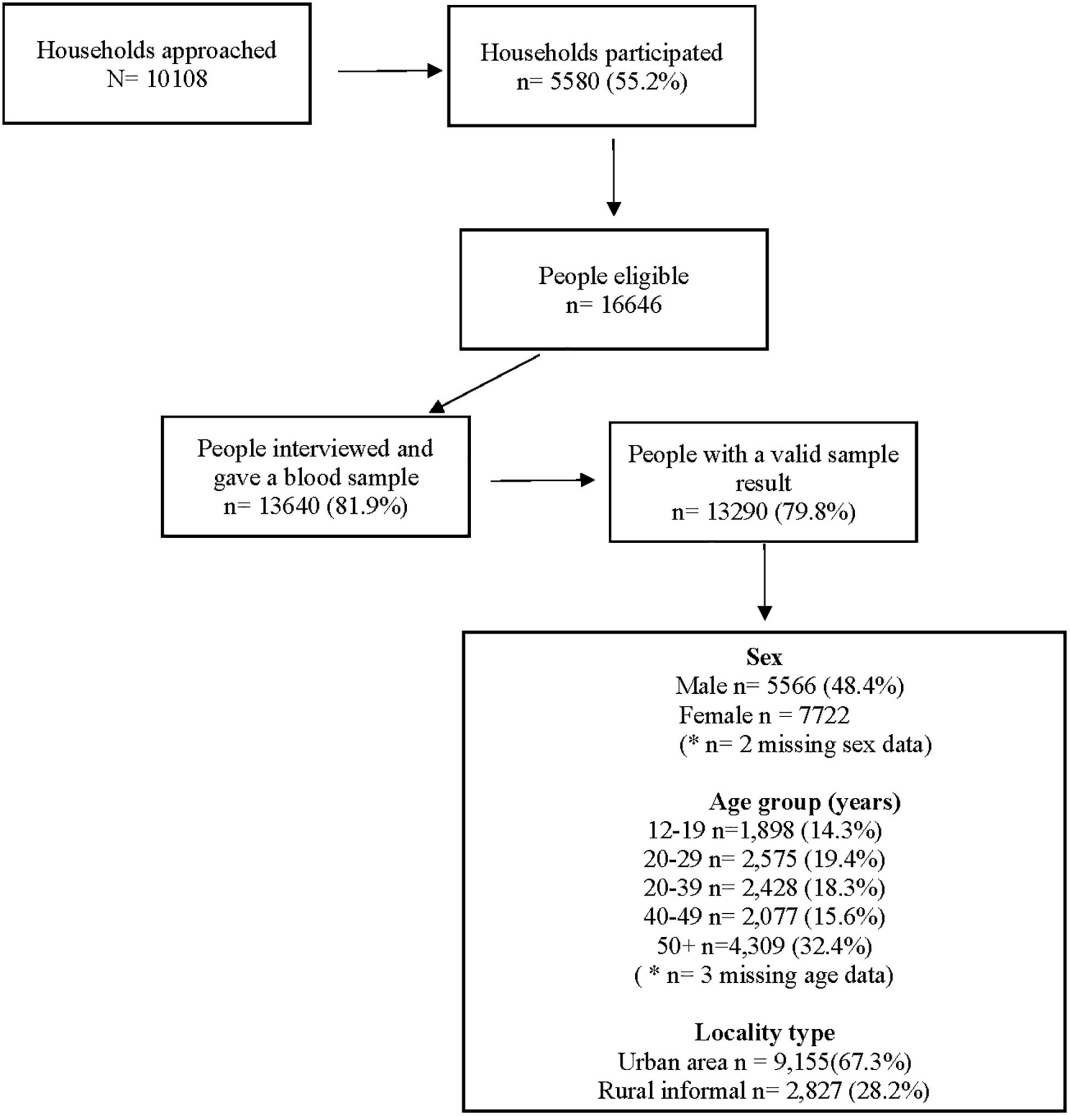
Survey participation.

Fig 1 shows the survey participation and key sociodemographic characteristics of survey participants. The household participation rate was 55.2% and within the households, the individual participation rate was 79.8%, with 13,290 participants having a valid test result included in the analysis. About half of the participants were females (51.6%). The median age was 33 years, and the interquartile range (IQR) 23–46 years with 14.3% aged 12–19 years old. The majority of participants (67.3%) lived in urban areas. 2,332/13,290 (17.51%) of participants provided data on the number of people living in their households and among these 418 (17.3%, weighted %) lived alone, while 1,034 (42.1%, weighted %)) lived with more than three household members Among the 11,687 participants who provided data on employment status 6,212 (54.7%, weighted %) were unemployed, and 308 (3.8%, weighted %) were fulltime students.

### SARS COV-2 seroprevalence and associated factors

**Fig 2:**
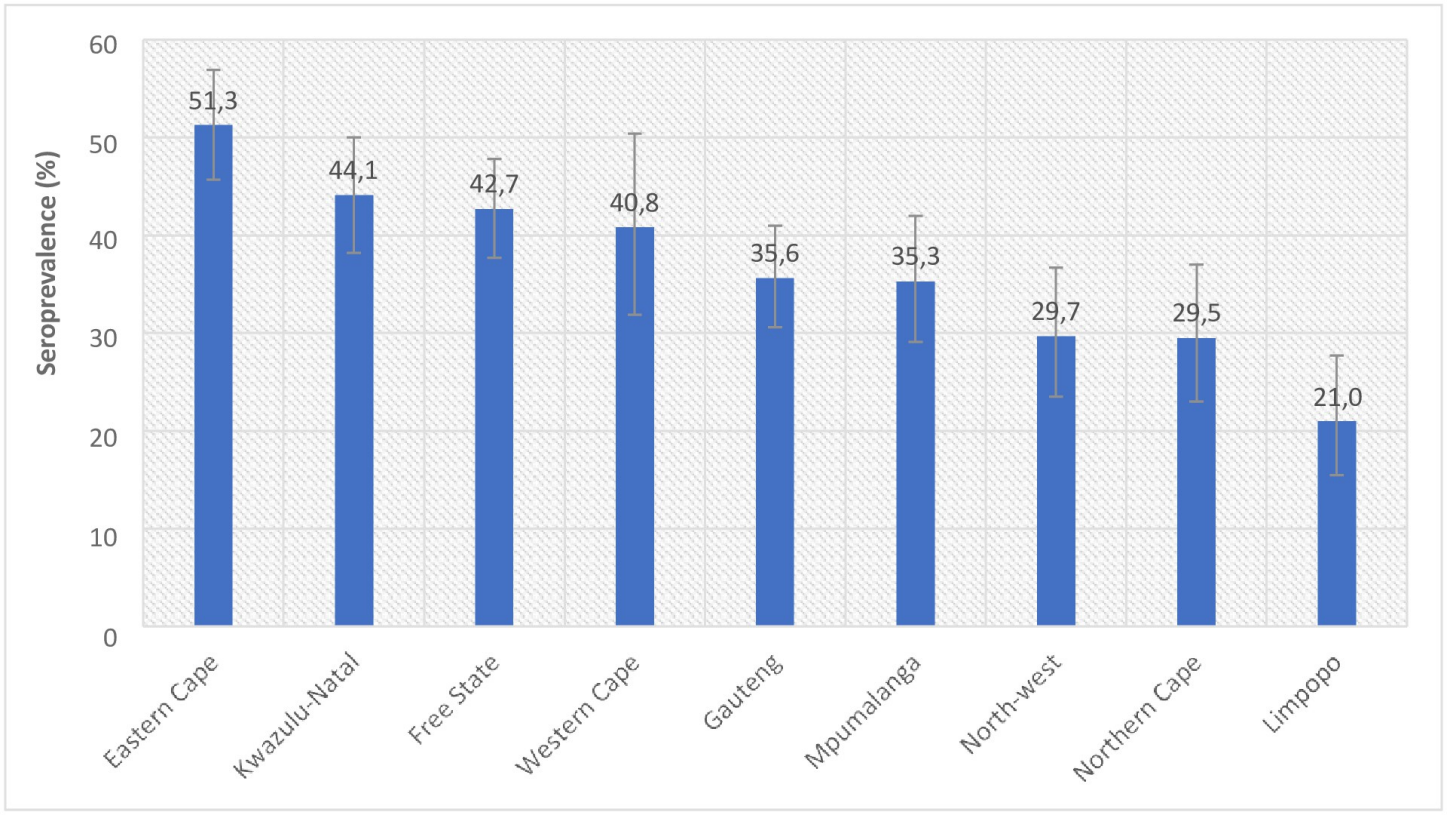
SARS COV-2 seroprevalence by province, among people 12 years and older (South Africa, 2021).

Overall, SARS-CoV-2 seropositivity over the survey period was estimated at 37.8% (95% CI 35.4–40.4) nationally, with substantial variation across the different provinces (Fig 2). Seroprevalence was highest in the Eastern Cape (51.3%, (95% CI: 45.7–56.9) and lowest in Limpopo province (21.0%, 95% CI: 15.5–27.7). The overall seroprevalence estimate translates to approximately 17 million people aged 12 years and older who had been infected at the time of the survey.

Seropositivity varied significantly by sex, and age group with no differences by employment status, household size and locality type. (Table 1). In bivariate logistic regression models SARS-CoV-2 seropositivity was significantly associated with sex and age:- with women more likely to be seropositive compared to men (OR 1.3, 95% CI 1.1–1.5), and those 50 years and older were less likely to be positive compared to the youth aged 12–19 years (OR 0.7, 95%CI 0.6–0.8) (Table 1).

**Table 1:** SARS-CoV-2 seroprevalence and association with socio-and demographic characteristics among people 12 years and older (South Africa, 2021).

Variables	SARS-CoV-2 seroprevalence				Bivariate models			
	N	%	95% CI	p-value	OR	95% CI		p-value
**Sex**								
Man	5,566	35.1	32.2–38.8	0.001	1			
Woman	7,722	40.7	37.7–43.8		1.3	1.1	1.5	0.001
**Age group in years**								
12–19	1,898	40.5	35.9–45.2	0.003	1			
20–29	2,575	40.0	36.9–45.1		1.0	0.8	1.3	0.856
30–39	2,428	38.2	34.3–42.2		0.9	0.7	1.2	0.444
40–49	2,077	38.7	33.8–43.7		0.9	0.7	1.2	0.538
50+	4,309	31.6	29.0–34.3		0.7	0.6	0.8	0.001
**Employment status**								
Unemployed	7,189	36.9	34.2–39.7	0.134	1			
Employed	4,424	39.6	36.2–43.1		1.1	1.0	1.3	0.134
**Household size**								
1	418	28.6	20.6–38.3	0.723	1			
2–3	880	27.1	20.7–34.6		0.9	0.5	1.6	0.787
4+	1,034	30.5	25. 8–35.7		1.1	0.7	1.8	0.710
**Locality type**								
Urban areas	9,155	39.2	36.0–42.5	0.050	1			
Rural informal/tribal areas	2,827	34.3	30.3–38.6		0.8	0.6	1.0	0.077
Rural/farm areas	1,308	44.0	38.5–49.7		1.2	0.9	1.6	0.140

Tables 2 & 3 show SARS-CoV-2 seroprevalence and association with socio-behavioural factors, COVID-19 symptoms, and comorbidities. SARS-CoV-2 seropositivity was significantly associated with attending an event or gathering with no statistically significant differences in associations with other behavioural characteristics, contact history, and sociodemographic factors including household size and employment status (Table 2). When analyzed by comorbidities, seropositivity was significantly associated with those who had diabetes and those with HIV infection (Table 3).

**Table 2:** SARS-CoV-2 seroprevalence and association with socio-behavioural factors, symptoms and testing among people 12 years and older (South Africa, 2021).

Variables	SARS-CoV-2 seroprevalence				Bivariate models			
	N	%	95% CI	p-value	^^OR	95% CI		p-value
**Left province/ village/suburb/ township in the past 7days**								
Yes	2,492	38.8	34.6–43.1		1.1	0.9	1.5	0.412
**Attended an event or gathering**								
Yes	1,409	42.6	37.4–48.1		1.3	1.1	1.7	0.017
**Close contact with people outside your home in the past 7days**								
Yes	4,356	37.8	34.3–41.4		1.1	0.8	1.4	0.616
**Number of close contacts the last time away from home**								
1–3 people	587	38.7	29.1–49.2	0.131	1			
4–10 people	1,499	31.7	27.8–35.9		0.7	0.5	1.1	0.175
10–20 people	1,346	42.0	37.2–46.9		1.1	0.7	1.8	0.551
More than 20 people	2,222	37.2	32.5–42.0		0.9	0.6	1.5	0.794
**Contact with suspected/confirmed COVID-19 infected person**								
No	9,369	38.5	35.6–41.4	0.057	1			
Yes	551	46.2	38.5–54.1		1.4	1.0	1.8	0.056
Do not know	2,883	35.2	31.0–39.6		0.9	0.7	1.1	0.181
**Place of contact with someone with suspected/ confirmed COVID-19 infection**								
Health Care Setting	25	37.5	16.2–65.5	0.727	1			
Family Setting	317	46.0	36.0–56.3		1.4	0.4	4.6	0.578
Workplace Setting	113	45.9	27.9–65.1		1.4	0.3	5.6	0.636
Public Transport setting	13	18.7	4.4–53. 5		0.4	0.1	2.3	0.289
In a retail store	9	34.4	8.0–76.0		0.9	0.1	7.2	0.890
Other (Specify)	54	54.4	31.1–75.8		2.0	04	8.7	0.372
**Any symptoms in the past 14days**								
Yes	740	34.5	27.8–41.9	0.348	0.9	0.6	1.2	0.345
**Any symptoms in the past 3 months**								
Yes	748	40.9	33.6–48.7	0.422	1.1	0.8	1.6	0.423
**Ever tested for SARS-CoV-2 infection**								
Yes	1,456	40.8	35.5–46.6		1.1	0.9	1.4	0.259

^^reference categories are the No responses

**Table 3:** SARS CoV-2 seroprevalence and association with ^#^medical history and ^#^comorbidities among people 12 years and older (South Africa, 2021).

Variables	SARS CoV-2 seroprevalence				Bivariate models			
	N	%	95% CI	p-value	^^OR	95% CI		p-value
**Tuberculosis**								
Yes	73	39.2	22.4–58.9	0.904	1.1	0.5	2.3	0.904
**Moderate to severe asthma**								
Yes	176	33.0	19.8–49.7	0.523	0.8	0.4	1.6	0.524
**Other chronic lung disease**								
Yes	27	34.6	15.1–61.1	0.789	0.9	0.3	2.6	0.789
**Hypertension/ high blood pressure**								
Yes	1,957	371	32.4–42.1	0.693	1.0	0.8	1.2	0.693
**Diabetes**								
Yes	784	30.8	24.9–37.4	0.031	0.7	0.5	1.0	0.033
**Cancer (that is not in full remission)**								
Yes	30	18.6	6.6–43.0	0.092	0.4	0.1	1.2	0.110
**HIV**								
Yes	549	44.8	38.0–51.9	0.047	1.4	1.0	1.8	0.048
**Cardiovascular conditions**								
Yes	151	33.3	18.1–53.0	0.733	0.8	0.4	1.8	0.608
**Other chronic conditions** ^##^								
Yes	20	21.5	7.4–46.7	0.159	0.4	0.1	1.5	0.172

^#^ Self-reported;

^##^Lung and kidney conditions;

^^reference categories are the No responses

**Fig 3:**
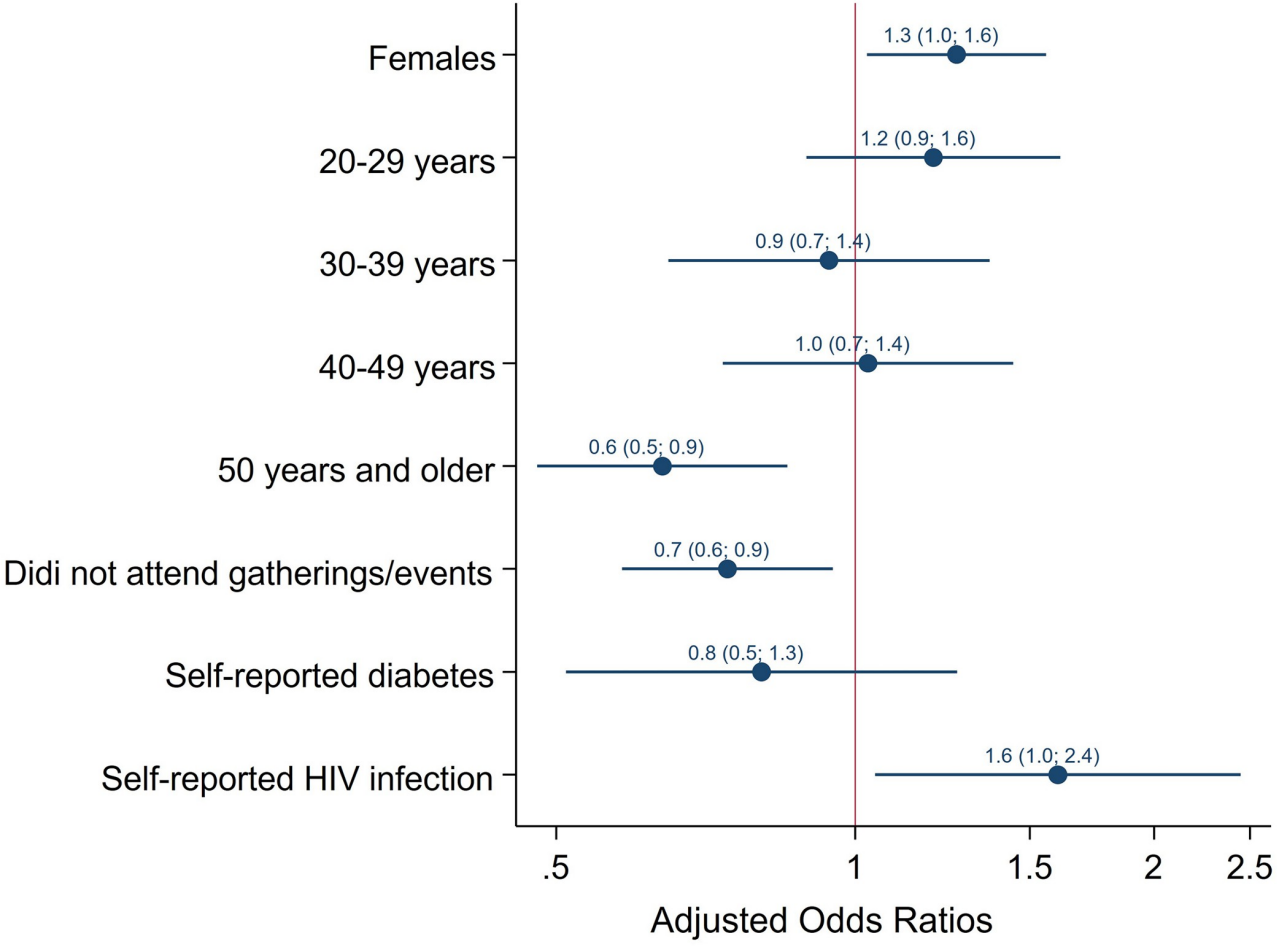
Multivariate logistic regression model of factors associated with SARS-CoV-2 seropositivity among people 12 years and older (South Africa, 2021).

**Fig 4:**
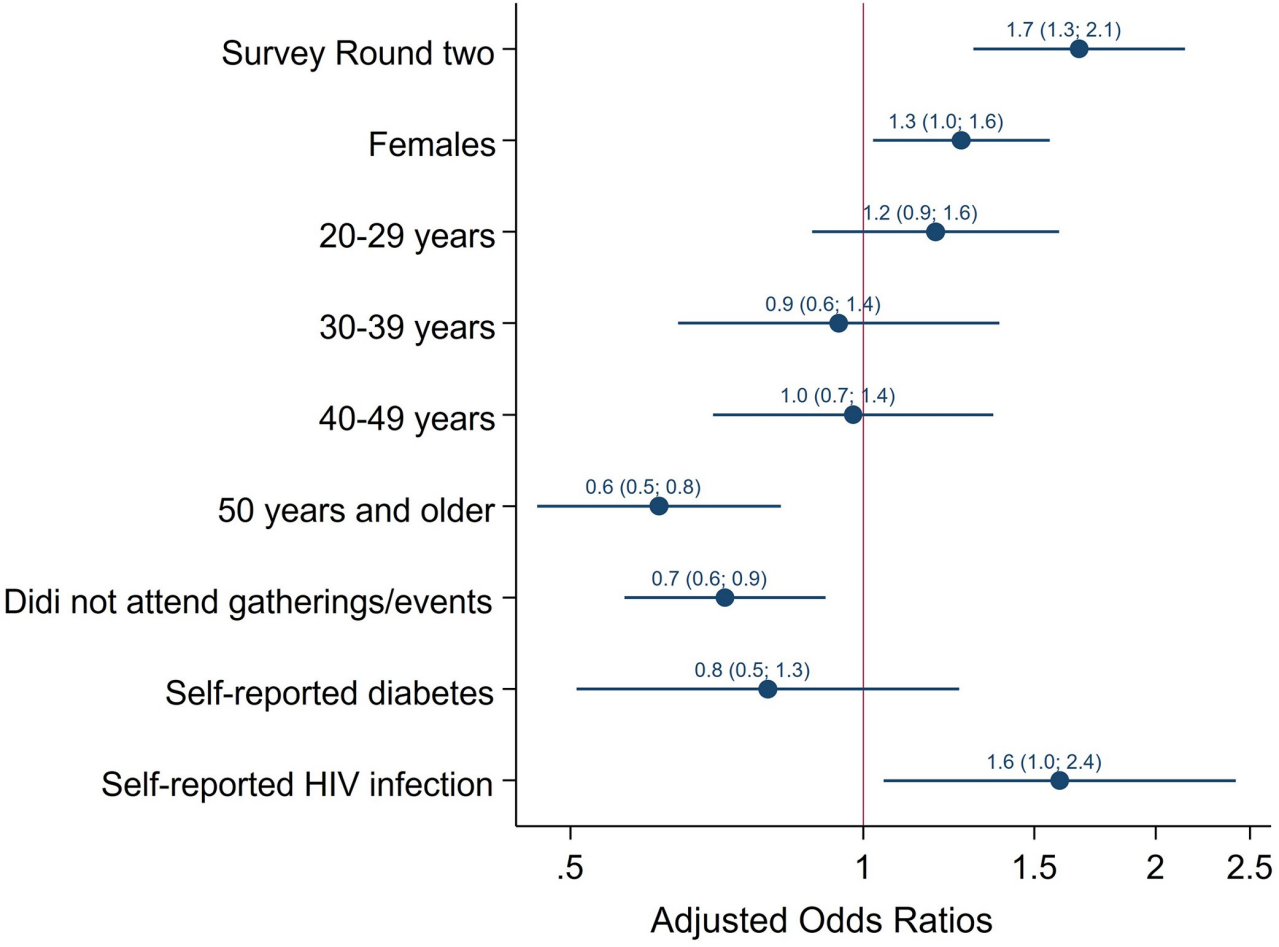
Multivariate logistic regression model of factors associated with SARS-CoV-2 seropositivity among people 12 years and older (South Africa, 2021).

In the final multivariate logistic regression models (Fig 3) the odds of SARS-CoV-2 seropositivity were significantly higher in women than in men [aOR = 1.3 (95% CI: 1.0–1.6), p = 0.017), and in those who reported HIV infection [aOR = 1.6 (95% CI: 1.0–2.4), p = 0.030]. The odds were significantly lower among those 50 years and older compared to youth 12–19 years old [aOR = 0.6 (95% CI: 0.5–0.9), p = 0.003]. and in those who did not attend events or gatherings compared to those who did [aOR = 0.7 (95% CI 0.6–0.9), p = 0.017]. When including survey round as a covariate in the multivariate regression analysis (Fig 4), the odds of SARS-CoV-2 seropositivity were significantly higher among those enrolled in round two of the survey [aOR = 1.7 (95% CI 1.3–2.1), p <0.001], in women than in men [aOR = 1.3 (95% CI: 1.0–1.6), p = 0.030), and those who reported HIV infection [aOR = 1.6 (95% CI: 1.0–2.4), p = 0.029]. The odds were significantly lower in those 50 years and older compared to adolescents 12–19 years old [aOR = 0.6 (95% CI 0.5–0.8), p = 0.001] and in those who did not attend events or gatherings [aOR = 0.7 (95% CI 0.6–0.9, p = 0.007].

## Discussion

In this nationally representative population-based household survey of SARS-CoV-2 antibodies in South Africa seroprevalence was estimated at 37.8% (95% CI 35.4–40.4 during Waves 2 and part of Wave 3 (November 2020 to February 2021, Wave 2, and May to September 2021, Wave 3) of the epidemic in 2021 [22]. This translates to an estimated 17 million infections in this population at this time. A COVID-19 wave was defined as a time period when the COVID-19 weekly incidence (new cases) was ≥30 confirmed cases per 100 000 persons until the weekly incidence was below 30 cases per 100 000 persons [22]. Seroprevalence varied substantially across the nine provinces and was highest in the Eastern Cape and lowest in the Limpopo province. On 3 July 2021, a few weeks after the conclusion of this study South Africa had reported a cumulative total of 2,062,896 laboratory-confirmed cases, with the Eastern Cape province having recorded 209,462 cases and Limpopo province 81,909 cases accounting for 10.2% and 4.0% respectively of the cumulative laboratory-confirmed cases in the country at the time [23]. This is comparable to but slightly lower than our survey findings where the Eastern Cape and Limpopo provinces accounted for 14.6% (2, 459,191) and 5.4% (919,932) respectively of the estimated 17 million survey infections. These results add to literature highlighting the importance of population-based seroprevalence estimates in understanding the true extent of infections, identifying populations most exposed to COVID-19, and also provide some information about possible population level humoral immunity at the time [12]. Population level seropositivity estimates are not subject to bias from unequal availability, distribution or uptake of testing services, and are thus important for informing the implementation of appropriate at scale public health mitigation measures and policies [12].

In the final multivariate regression models increased odds of SARS-CoV-2 seropositivity was associated with being a woman. Subnational and donor studies conducted in South Africa did not find differences in seroprevalence by sex and reviews by Lai et al., and Bergeri et al., also concluded that seropositivity did not differ substantially by sex [11, 12]. However, the number of reported cases based on testing data for South Africa reported on 3 July showed a greater number of cases in women than in men [23]. In COVID-19 hospital admissions data for South Africa over the period March 2020 and 8 January 2022, incidence was higher in women (526.8/100,000 persons) than in men (417.0/100,000 persons) [24]. Higher infections in women than men in South Africa have been attributed to women being more represented in occupations and roles that placed them at greater risk of exposure both within and outside the home [23]. Policy responses should therefore account for these differences in exposure.

We also found greater odds of seropositivity in people who reported being HIV positive contrary to findings of other surveys in South Africa [14, 15]. While there is no clinical evidence that people living with HIV (PLHIV) have a higher risk of infection with SARS-CoV-2 when compared with HIV-negative people, HIV was shown to be a risk factor for severe COVID-19 and mortality, while ART and viral load suppression are associated with reduced risk for poor outcomes [25, 26]. Therefore PLHIV should continue to be monitored and be especially targeted for COVID-19 vaccination in combination with efforts for retention on antiretroviral treatment and viral suppression.

Older people (50 years and older) were less likely to be infected than younger people (12–29 years old). The relationship between SARS-CoV-2 infection and age varies across settings. A seroprevalence survey in Gauteng province of South Africa (November 2020, January 2021) found no differences in seroprevalence by age, while in Zambia seroprevalence was lowest among those 15–19 years old in data collected between October 2020 and March 2021 [15, 27]. A systematic review and meta-analysis of standardized population-based studies from January 2020 to April 2022, reported that children 0–9 years old and adults 60 years and older were at lower risk of seropositivity than adults 20 to 29 years old (p < 0.001 and p = 0.005, respectively), with relatively similar levels of infections in those aged 10–19 years and those 30–39, 40–49 and 50–59 years old, and in France seroprevalence was also higher in those aged 20–29 years old than in those 50-years and older [12, 28]. Our results indicate that many adolescents had asymptomatic or mild diseases [29, 30], since cases detected by PCR were mainly in middle aged and older people at the time our survey ended [23]. During and soon after this survey, the guidelines were to test when symptomatic and following contact with someone diagnosed with COVID-19 [31]. The higher odds of seropositivity in adolescents may be attributed to adolescents having more contacts than adults through school and other social circles and also partly to the fact that adults were more likely to adhere to masking and social distancing because they felt vulnerable [29, 32]. Therefore adolescents could have contributed to spreading the virus [32]. These findings highlight the importance of providing clear and consistent COVID-19 control and prevention messages targeting and tailored to all age groups. Before vaccination roll out, events/gatherings were confirmed as infection super spreaders due to crowding, and close and prolonged frequent interactions between people, representing a conducive environment for transmission of SARSCoV-2 [33]. Our findings concur with this:- we found significantly lower odds of seropositivity among those who had not attended events or gatherings.

The variations in seroprevalence by sociodemographic and behavioural characteristics, and comorbidities highlight the need for continued population based national and local level surveillance of seroprevalence to estimate the burden, monitor the dynamics and understand the risks and characterize the most vulnerable groups to better inform and adapt additional interventions as COVID-19 vaccination coverage also expands and given that new strains may circulate [11, 12, 34]

This study had some limitations. Firstly, the estimates should be interpreted with caution since survey data was collected over a prolonged time period (4–5 months) in a rapidly changing epidemic. Secondly, the household participation was low (55.2%). However individual level participation was high (79.8%). Thirdly, antibodies against the SARS-CoV-2 virus wane over time, hence cumulative incidence may have been underestimated [35–37]. Furthermore, there are differences in the performance of various tests when used for serosurveillance and this may have impacted the estimates, although the Roche ElecsysAnti-SARS-CoV-2 assay we used has previously been shown to maintain seroreactivity and high sensitivity over time [21, 38]. The study excluded children younger than 12 years, and therefore is not representative of the entire South African population. However, data were weighted and benchmarked to the national population aged 12 years and older and the weights were also adjusted for clustering. Self-reported information collected from interviews is subject to social desirability bias and could have impacted associations for some characteristics and factors. Finally, data on comorbid conditions may be underestimated since we collected data in households and thus likely excluded those hospitalised who were more likely to have comorbid conditions that placed them at greater risk of infection and severe disease. Despite these limitations, this is the first nationally representative household SARS-CoV-2 seroprevalence survey in South Africa that included diverse population groups in all provinces and locality types in the country. It was conducted before widespread vaccination against SARS-CoV-2 and thus presents a national baseline assessment of the extent of SARS-CoV-2 exposure and infection before vaccination.

## Conclusion

This study adds to data on seroprevalence in low- and middle-income countries to better understand the global extent and dynamics of SARS-CoV-2 infection over time.

Women and adolescents and were more likely to be infected. While testing had already indicated a higher burden of infection in women, this survey highlighted the burden and risk in younger people indicating the need for age-appropriate prevention messaging and the urgency to vaccinate younger people since they can spread infections to members of their circles and households who may be at greater risk of severe disease and poor outcomes. The study also showed the national epidemiological picture of infections and heterogeneity by geographic area, indicated the potential extent of under-ascertainment of cases based on PCR and antigen testing, and affirmed the risks of events/gatherings for transmission of COVID-19, supporting regulations governing these activities at different time periods of the epidemic.
